# The validation of the Hungarian version of the ID-migraine questionnaire

**DOI:** 10.1186/s10194-018-0938-z

**Published:** 2018-11-12

**Authors:** Éva Csépány, Marianna Tóth, Tamás Gyüre, Máté Magyar, György Bozsik, Dániel Bereczki, Gabriella Juhász, Csaba Ertsey

**Affiliations:** 1 Szentágothai János Doctoral School of Neurosciences, Semmelweis University, Üllői u. 26, Budapest, 1085 Hungary; 2 Department of Neurology, Vaszary Kolos Hospital, Petőfi Sándor u. 26-28, Esztergom, 2500 Hungary; 3 Department of Neurology, Semmelweis University, Balassa u. 6, Budapest, 1083 Hungary; 4 SE-NAP2 Genetic Brain Imaging Migraine Research Group, Semmelweis University, Nagyvárad tér 4, Budapest, 1089 Hungary; 5 Department of Pharmacodynamics, Faculty of Pharmacy, Semmelweis University, Nagyvárad tér 4, Budapest, 1089 Hungary

**Keywords:** ID-migraine questionnaire, Hungarian version, Validity features, Migraine

## Abstract

**Background:**

Despite its high prevalence, migraine remains underdiagnosed and undertreated. ID-Migraine is a short, self-administrated questionnaire, originally developed in English by Lipton et al. and later validated in several languages. Our goal was to validate the Hungarian version of the ID-Migraine Questionnaire.

**Methods:**

Patients visiting two headache specialty services were enrolled. Diagnoses were made by headache specialists according to the ICHD-3beta diagnostic criteria. There were 309 clinically diagnosed migraineurs among the 380 patients. Among the 309 migraineurs, 190 patients had only migraine, and 119 patients had other headache beside migraine, namely: 111 patients had tension type headache, 3 patients had cluster headache, 4 patients had medication overuse headache and one patient had headache associated with sexual activity also. Among the 380 patients, 257 had only a single type headache whereas 123 patients had multiple types of headache. Test-retest reliability of the ID-Migraine Questionnaire was studied in 40 patients.

**Results:**

The validity features of the Hungarian version of the ID-Migraine questionnaire were the following: sensitivity 0.95 (95% CI, 0.92–0.97), specificity 0.42 (95% CI, 0.31–0.55), positive predictive value 0.88 (95% CI, 0.84–0.91), negative predictive value 0.65 (95% CI, 0.5–0.78), missclassification error 0.15 (95% CI, 0.12–0.19). The kappa coefficient of the questionnaire was 0.77.

**Conclusion:**

The Hungarian version of the ID-Migraine Questionnaire had adequate sensitivity, positive predictive value and misclassification error, but a low specificity and somewhat low negative predictive value.

## Background

Migraine is a common disease, which affects 14% of the population, and 18% of women [1] globally. In the USA, its lifetime prevalence is 25% in women [2]. It affects mainly the active, working, young adult population [3]. In Hungary, only one population based headache epidemiology study has been made to date [4]. This study reported 67% lifetime prevalence for any kind of headache: the one-year prevalence of migraine without aura was 7.6%, and the one-year prevalence of migraine with aura was 2%. Only 43% of migraineurs had ever consulted any physician because of their headache, and 15% of patients missed school or work because of their headache in the previous year.

According to the report of Global Burden of Disease studies, migraine is the third cause of disability in 15–49 years old men and women [5]. The disability, caused by migraine, affects many aspects of life, and leads to both physcial and emotional impairment [6]. In one Swedish study, researchers found that in migrainous patients, the health-releated quality of life is significantly worse, not only during the migraine attacks, but also between attacks, compared to healthy controls. In another study, 65% of migraine patients reported some degree of absenteeism from their workplace or school due to their headache [6]. The indirect costs of migraine are considerable: in the USA alone, approximately 13 billion dollars are spent each year for migrainre-related absenteeism from workplace and reduced ability to work [7]. Despite migraine’s serious negative effects on the individual, less than half of the patients ever recieve a medical diagnosis of headache [8, 9]. Furthermore, migraine is suboptimally treated, with only one-third receiving some kind of migraine-specific medication [10]. There are several factors of migraine being underdiagnosed and undertreated. The most important factor is that many migraine patients – even those with quite strong headaches – do not consult their doctors because of their headache, and therefore do not receive the diagnosis of migraine [8]. In the UK, 4.4% of the population would see a general practitioner because of a headache problem in a given year, 34% of whom (ie. 1.5% of the population) would be prescribed a migraine medication (acute or prophylactic), while only 2.1% of those who consult would be referred to a neurologist [11]. This compares to a 14.3% one-year prevalence of migraine in the UK [12]: the reason for the low consultation rates is not self-evident [11]. The severity of the attack may not be a decisive factor in consulting: an American survey found that 61% of those who had never consulted reported severe or very severe pain and 67% reported severe disability or the need for bed rest during their migraines [13]. A further difficulty in diagnosing migraine may be the duration of the doctor-patient meeting, which may not be enough to discuss the characteristics of the headaches. Furthermore, a number of primary care physicians may not have an adequate knowledge about headaches [14], and the IHS criteria are excessively complex and time-consuming for routine application in primary care [15, 16].

In order to facilitate the detection of migraine in primary care, Lipton et al. (2003) developed a brief, self-administered questionnaire, the ID-Migraine [17]. The questionnaire contains two pre-screening questions, one asking about headache-related disability, the other asking whether the patient would like to consult a doctor because of the headache. This is followed by three screening questions pertaining to the previous three months. These screening questions ask about headache-related disability, nausea and sensitivity of light. The ID-Migraine indicates migraine if a patient answered “yes” at least to two of the three screening questions. In the original study, the sensitivity of the ID-Migraine Questionnaire was 0.81, the specificity was 0.75, and the positive predictive value was 0.93. Test-retest reliability was good, with a kappa of 0.68. The ID-Migraine Questionnaire was therefore found to be a valid, reliable, and easy-to-use screening instrument to detect migraine, for patients presenting in primary care. The authors emphasized that the ID-Migraine Questionnaire is not a diagnostic instrument by itself, so a thorough evaluation of patients is necessary to make the diagnosis of migraine. Subsequently, the questionnaire was validated in Italian [18], Portuguese [19] and Turkish [20] languages with adequate results (Table 1). The questionnaire has been used in many specialty fields to screen migraine, not only in primary care [16, 21], but also in headache centers and neurology clinics [18, 19, 22]. Moreover, the questionnaire was successfully used for screening migraine patients in the emergency department [23], in a temporomandubular and orofacial pain clinic [24], and also in opthalamic and ear, nose and throat clinics [25]. The ID-Migraine Questionnaire proved to be reliable not only in adults, but also among adolescents [26]. It was also used in large-scale studies of migraine epidemiology [21] and genetic studies [27].

**Table 1:** Validation results of the ID-Migraine questionnaire in different languages

	Location of the research	Sensitivity (95% CI)	Specificity (95% CI)	PPV (95% CI)	NPV (95% CI)
English,2003[17]	primary care	0.81 (0.77 to 0.85)	0.75 (0.64 to 0.84)	0.93 (0.90 to 0.96)	NI
Italian, 2007[18]	headache centers	0.95 (0.91 to 0.98)	0.72 (0.62 to 0.82)	0.88 (0.82 to 0.93)	0.87 (0.78 to 0.95)
Turkish, 2007[19]	neurology outpatient clinics	0.92 (NI)	0.63 (NI)	0.72 (NI)	0.88 (NI)
Portuguese, 2008[20]	headache outpatient clinics	0.94 (0.87 to 0.97)	0.60 (0.46 to 0.73)	0.80 (0.71 to 0.87)	0.85 (0.70 to 0.94)

*NI*=no information, *PPV* = positive predictive value, *NPV* = negative predictive value, *CI* confidence interval

Note: NPV was not reported in the English study, classification errors were not reported in any of the studies, and no confidence intervals were reported in the Turkish study

In this study we present the validity features of the Hungarian version of the ID-Migraine Questionnaire.

## Methods

Patients between 18 and 65 years of age, presenting at the Headache Service of the Department of Neurology, Semmelweis University or the Headache Service of Esztergom Hospital, and reporting two or more headaches in the previous 3 months were involved. Both Services worked with the same methology. In order to include at least 300 patients in the study (ie. 100 patients per questionnaire item, which is a widely accepted and conservative way of assuring an adequate sample size in validation studies [28] we involved all patients visiting these Services in a two-year period who were willing to participate and gave informed consent to processing their results. The study protocol had been approved by the ethics committee of Semmelweis University. Patients completed the questionnaire at the occasion of their medical visit. Most of the patients filled in the questionnaire before the medical visit, ie. while they were waiting to be seen by the neurologist, who, on the other hand, did not use the questionnaire to ascertain the diagnosis that was based on the interview with the patient. A minority of the patients filled in the questionnaire while they were waiting for their written documentation, ie. after the medical visit. The patients also completed a 9-item Hungarian migraine screener (the MDX questionnaire), developed and validated by our group [29], which was used to collect information about the clinical characteristics of their headaches in more detail, but was not included as a reference tool in the validation process of ID-Migraine. All the patients underwent detailed internal medicine and neurological examination. The gold standard was the neurologists’ clinical diagnosis, according to the International Classification of Headache Disorders (ICHD3-β). As in the original English version [17], the Hungarian version of the ID-Migraine was considered positive for migraine if a patient answered “yes” at least to two of the three screening questions. The responses to the ID-Migraine Questionnaire were then compared with the clinical diagnosis of migraine: if a patient had a diagnosis of migraine, she/he was considered a migraineur regardless of having other headaches beside migraine or not. Based on the primary diagnosis, the questionnaire’s sensitivity, specificity, positive predictive value (PPV), negative predictive value (NPV), and classification error were calculated. These parameters were calculated for the individual items of the ID-Migraine Questionnaire, as well. Based on previously reported validation studies [18, 19] we also calculated these values in subgroups of patients according to sex, age (equal or below 44 years and above 44 years) and disease duration (equal or below 12 years and above 12 years) in order to have a more thorough vision of the Hungarian version’s performance.

In addition, to evaluate the characteristics of the Hungarian ID-Migraine Questionnaire the receiver operating curve (ROC) was constructed among the 380 patients with different sensitivity (true positive rate) and 100-specificity (false positive rate) values according to the minimum number of positive answers to the ID-Migraine Questionnaire (0, 1, 2, and 3 positive answers).

Among the 380 headache sufferers, 40 patients completed the ID-Migraine Questionnaire twice, the second time was also during a follow-up visit. Test-retest reliability, using the Cohen’s Kappa, was calculated in these 40 patients. The following values of Cohen’s Kappa were used to evaluate the level of agreement [30]: < 0: no agreement; 0.0–0.20: slight agreement; 0.21–0.40: fair agreement; 0.41–0.60: moderate agreement; 0.61–0.80: substantial agreement; 0.81–1.0: perfect agreement.

We used an Excel spreadsheet for data input, and an online statistical package (VassarStats, http://vassarstats.net/) to calculate the ID-Migraine’s validity features (sensitivity, specificity, PPV, NPV), the confidence intervals thereof, misclassification error and test-retest reliability.

## Results

A total of 380 patients completed the Hungarian version of the ID-Migraine Questionnaire. Among the 380 patients, 80% were female and 20% were male. The median age was 36 years, the interquartile range was 19.8 years. The median disease duration was 10 years, the interquartile range was 16 years.

Table 2 summarizes the clinical headache diagnoses among the 380 patients. The number of clinically diagnosed migraineurs was 309; among them, 190 had only migraine, whereas 119 patients had another headache diagnoses beside migraine. The total number of non-migraine patients was 71; the primary diagnosis was tension type headache (TTH) in 45 patients, cluster headache in 19 patients, and other headache in 7 patients. Among the 380 patients, 257 had only one type headache, namely: 190 patients had only migraine, 44 had only TTH, 16 had only cluster headache and 7 had only other type of headache. The other 123 patients had more than one type of headache at the time of the study.

**Table 2:** Clinical headache diagnoses among the 380 patients who completed the ID-Migraine questionnaire

Primary diagnosis	Number	Secondary diagnosis	Number
Migraine	309 (251 episodic and 58 chronic)	none	190
		tension type headache	111
		cluster headache	3
		medication overuse headache	4
		headache associated with sexual activity	1
Tension type headache	45 (12 episodic and 33 chronic)	none	44
		headache associated with sexual activity	1
Cluster headache	19 (18 episodic and 1 chronic)	none	16
		tension type headache	2
		SUNCT syndrome	1
Other headache	7	none	7

*MOH* medication overuse headache, *SUNCT* short-lasting unilateral neuralgiform headache with conjunctival injection and tearing

Among the 380 patients, 334 had a positive ID-Migraine score; 293 of them had a clinical diagnosis of migraine. Among the 45 patients, clinically diagnosed with TTH, 23 had a positive ID-Migraine score, as did 16 of the 19 patients, whose clinical diagnosis was cluster headache. Table 3 contains the number of positive ID-Migraine Questionnaires in the clinical headache groups.

**Table 3:** The number of ID-Migraine Questionnaire positive patients in clinically diagnosed headache groups

Clinically diagnosed patients	ID-Migraine Questionnaire positive patients
total sample size: *n* = 380	*n* = 334
migraine group: *n* = 309	*n* = 293
tension type group: *n* = 45	*n* = 23
cluster type group: *n* = 19	*n* = 16
other headache group: *n* = 7	*n* = 2

**Fig. 1:**
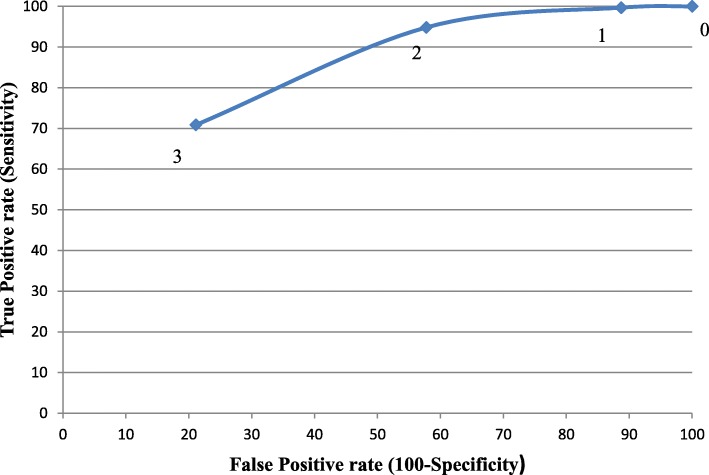
The receiver operating curve of the ID-Migraine Questionnaire in the study population. The curve shows the sensitivity and specificity values according to the minimum number of positive answers for the ID-Migraine Questionnaire. Thresholds were determined according to the minimum number of positive answers to the ID-Migraine Questionnaire (0,1, 2 and 3 positive answers). 0: sensitivity: 1.00 (95% CI; 0.98–1.00); specificity: 0.00 (95% CI; 0.00–0.06). 1: sensitivity: 0.997 (95% CI; 0.98–1.00); specificity: 0.11 (95% CI; 0.05–0.22). 2: sensitivity: 0.95 (95% CI; 0.92–0.97); specificity: 0.42 (95% CI; 0.31–0.55). 3: sensitivity: 0.71 (95% CI; 0.65–0.76); specificity: 0.79 (95% CI; 0.67–0.87)

Figure 1 shows the ROC curve with different cut off points (0, 1, 2, or 3 “yes” answere) to demonstrate the characteristics of the Hungarian ID-Migraine Questionnaire. To calculate validity measures we used the original cutoff value of at least two “yes” answers out of the three screening questions as reported by Lipton et al. [17].

Based on the whole sample (*n* = 380), the quality scores of the Hungarian version of the ID-Migraine Questionnaire were the following: sensitivity 0.95 (95%CI, 0.92–0.97), specificity 0.42 (95%CI, 0.31–0.55), positive predictive value (PPV) 0.88 (95%CI, 0.84–0.91), negative predictive value (NPV) 0.65 (95%CI, 0.5–0.78), misclassification error 0.15 (95%CI, 0.12–0.19).

Fourty of the 380 patients also completed the questionnaire during a follow-up visit. In this sample, the clinical diagnoses were as follows: 31 patients had migraine, 6 had TTH, 2 had cluster headache, and one had other (cervicogenic) headache. Two of the patients’ clinical migraine diagnoses changed between the first and second ID-Migraine Questionnaire assessments: one had migraine as the initial diagnosis and TTH at follow-up, the other had TTH as the initial diagnosis and migraine at follow-up. The median interval between filling out the ID-Migraine Questionnaire for the first and second time was 90.5 days, the interquartile range was 475 days. At the time of the first completion, 36 of the 40 patients had a positive ID-Migaine Questionnaire. At the second time, 34 of the 40 patients had a positive test. The kappa coefficient of the questionnaire was 0.77, indicating a substantial agreement between the assesments. The overall percent of agreement was 0.95, the percent of positive agreement was 0.94.

Table 4 summarizes the quality scores for the separate items of the ID-Migraine Questionnaire using the data of 380 patients. All of the items (nausea, photophobia and disability) had high sensitivity and positive predictive value (> 0.8). We found the highest sensitivity for the disability (0.97), similarly to the Italian ID-Migraine Questionnaire. Nausea showed the highest positive predictive value (0.9). By contrast, we found signifcantly lower scores for the negative predictive value and specificity compared to sensitivity and positive predictive value.

**Table 4:** Quality scores separately for the items of the Hungarian version of the ID-Migraine Questionnaire (*n* = 380)

	Sensitivity (95% CI)	Specificity (95% CI)	PPV (95% CI)	NPV (95% CI)	Classification error (95% CI)
Nausea	0.86 (0.82–0.90)	0.59 (0.47–0.70)	0.9 (0.86–0.93)	0.49 (0.38–0.60)	0.19 (0.15–0.23)
Photophobia	0.83 (0.78–0.86)	0.58 (0.45–0.69)	0.89 (0.85–0.93)	0.43 (0.33–0.54)	0.22 (0.18–0.27)
Disability	0.97 (0.94–0.98)	0.15 (0.08–0.26)	0.83 (0.79–0.87)	0.52 (0.3–0.74)	0.18 (0.15–0.23)
ID-migraine positive (≥2 “yes”)	0.95 (0.92–0.97)	0.42 (0.31–0.55)	0.88 (0.84–0.91)	0.65 (0.5–0.78)	0.15 (0.12–0.19)

*PPV* = positive predictive value, *NPV* = negative predictive value, *CI* confidence interval

Table 5 presents the quality scores of the questionnaire in the clinically relevant subgroups, namely according to sex, age (equal or below 44 years and above 44 years) and disease duration (equal or below 12 years and above 12 years). While the sensitivity and specificity of the ID-Migraine was the same in female and male patients, the PPV was noticeably higher, whereas the NPV and misclassification error were noticeably lower in females than males. There were no other substantial differences between the subgroups.

**Table 5:** Quality scores of the Hungarian version of the ID-Migraine questionnaire in the clinically relevant subgroups

	N (%)	Sensitivity(95% CI)	Specificity (95% CI)	PPV (95% CI)	NPV (95% CI)	Classification error (95% CI)
Sex						
Women	304 (80%)	0.95 (0.92–0.97)	0.47 (0.30–0.65)	0.93 (0.90–0.96)	0.55 (0.36–0.73)	0.1 (0.07–0.14)
Men	76 (20%)	0.95 (0.81–0.99)	0.39 (0.24–0.56)	0.63 (0.49–0.75)	0.88 (0.60–0.98)	0.32 (0.22–0.44)
Age						
≤ 44 years	262 (69%)	0.96 (0.92–0.98)	0.44 (0.29–0.60)	0.89 (0.84–0.93)	0.69 (0.48–0.85)	0.13 (0.09–0.18)
> 44 years	118 (31%)	0.94 (0.86–0.98)	0.48 (0.27–0.69)	0.87 (0.78–0.93)	0.69 (0.41–0.88)	0.16 (0.10–0.24)
Duration of illness						
≤ 12 years	228 (60%)	0.96 (0.89–0.99)	0.41 (0.25–0.59)	0.82 (0.74–0.89)	0.78 (0.52–0.93)	0.18 (0.12–0.26)
> 12 years	152 (40%)	0.94 (0.85–0.98)	0.33 (0.09–0.69)	0.93 (0.84–0.97)	0.38 (0.10–0.74)	0.13 (0.06–0.21)

*PPV* = positive predictive value, *NPV* = negative predictive value, *CI* confidence interval

Given that the Hungarian version of the ID-Migraine had substantially lower specificity and NPV than the original and previous translations, we scrutinized those patients whose were diagnosed with nonmigraine headaches, especially whose ID-Migraine scores were positive, as was the case of 51% of TTH patients, 84% of cluster headache patients, and 29% of other headache patients, making use of the MDX questionnaire that contained the clinical characteristics of their headaches in more detail. Table 6 shows the clinical characteristics of these patients’ headaches. All patients in all diagnostic groups reported an at least moderate severity of headache, regardless of their ID-Migraine score. In the TTH group, patients with a positive ID-Migraine score had an average of 3.3 migrainous features, compared to 2.0 in their ID-Migraine negative peers. In the cluster headache group, patients with a positive ID-Migraine score had, on average, 4.0 migrainous features, compared to 0.3 in ID-Migraine negative patients. In the group of other non-migraine headaches, patients with a positive ID-Migraine score had an average of 3.5 migrainous features, whereas the ID-Migraine negative patients had 2.0.

**Table 6:** The self-reported headache characteristics of the non-migraine patients versus their ID-Migraine status

Clinical diagnosis	ID-Migraine	Number of patients	Worse with movement	Nausea	Vomiting	Photophobia	Phonophobia
Tension type headache	positive	23	14	17	1	12	9
	negative	22	12	1	0	2	8
Cluster headache	positive	16	9	9	8	12	10
	negative	3	0	0	0	0	1
Other headache	positive	2	1	1	0	1	2
	negative	5	2	0	0	0	3

Note: All patients reported an at least moderate severity of pain so this was not included In Table 6

In the TTH group, 23 patients (4 episodic and 19 chronic) had a positive ID-Migraine score. Based on the clinical characteristics available from the MDX questionnaires, 14 patients (1 episodic and 13 chronic) could be considered as having migraines as well, whereas 3 episodic and 6 chronic TTH patients did not meet the criteria of migraine. The difference between the distribution of suspected migraine and no migraine was not significant (Chi square test: *p* = 0.106).

In the cluster headache group, 16 patients (15 episodic and 1 chronic) had a positive ID-Migraine score. As all of these patients were followed up at the Dept. of Neurology, and their clinical documentation was available, we performed a retrospective chart review to ascertain the diagnosis. Based on the clinical characteristics (strictly unilateral attacks; presence of ipsilateral autonomic features; periodicity) the diagnosis of cluster headace was confirmed in all of them. However, as 84% of cluster headache patients had a positive ID-Migraine score and they were overrepresented in the sample, we also calculated the quality scores excluding these patients. In this calculation, sensitivity was 0.95 (95%CI: 0.92–0.97), specificity was 0.52 (95% CI: 0.38–0.66), PPV was 0.92 (95% CI: 0.88–0.95), NPV was 0.63 (95% CI: 0.47–0.77) and misclassification error was 0.11 (95%CI: 0.08–0.15).

Finally the two patients who had other non-migrainous headaches and a positive ID-Migraine score could be considered as having migraines as well, based on the characteristics of their headaches.

## Discussion

Our results demonstrated that the Hungarian version of the ID-Migraine Questionnaire is a reliable screening instrument for migraine based on data collected at specialist headache centres. The fact that all patients fully completed the questionnaire indicates that it is easy to understand and use, so it could be used as a screening tool in primary care, and also for research purposes. The sensitivity and positive predictive value of the Hungarian version of the ID-Migraine Questionnaire were quite similar to those of the original English version [17], with the Hungarian version having a higher sensitivity (0.95 vs. 0.81). The classification error (which had not been reported in the previous validation studies) was also acceptable. On the other hand, the specificity of the Hungarian version was markedly lower, and the negative predictive value was also somewhat lower than in the previous validation studies. It is important to note, that our sample was quite similar to other validation studies where the participants had also been recruited from headache centers [18–20, 29, 31–34].

All of the items (nausea, photophobia and disability) had high sensitivity and positive predictive value (> 0.8). This is in agreement with the Italian and Portugese versions of ID-Migraine Questionnaire [18, 19]. We found the highest sensitivity for the disability (0.97), similarly to the Italian ID-Migraine Questionnaire [18]. The nausea showed the highest positive predictive value (0.9), similarly to the portugese version of ID-Migraine Questionnaire [19], which supports the previous studies that headache-related nausea is an important accompaining symptom and has high impact on migraine [8, 35–39].

From the 309 clinically diagnosed migraine patients, 293 had positive ID-Migraine scores. This result supports the previous findings, that ID-Migraine Questionnaire has a high screening accuracy [17–19, 22, 25, 40–42].

In addition, the quality scores of the questionnaire showed no significant difference between clinically relevant subgroups, divided by sex, age and disease duration, similarly to the Portuguese and Italian versions of ID-Migraine Questionnaire [18, 19]. This suggests that the questionnaire may be used in the general population.

The cause of the lower specificity and NPV of the Hungarian version (0.42 vs 0.75 for the English version) may in part be due to the high number of false positive patients among the clinically diagnosed cluster headache and TTH patients. In particular, cluster headache was overrepresented in the sample (the prevalence of cluster headache patients in the sample was 5.0% versus a roughly 0.1% prevalence in the general population [43]. Sixteen of these 19 patients had a positive ID-Migraine score, which is not surprising considering the high incidence of nausea and photophobia occurring during cluster headache attacks, described first by Bahra et al. [44], and also corroborated by a Hungarian study [45] where the prevalence of nausea and photophobia during a cluster headache attacks were 43% and 68%, respectively. As shown in the Results section, eliminating the cluster headache patients resulted in a significant rise in the specificity, from 42% to 52%, while, interestingly, the NPV did not change much. Given the fact that the spontaneous occurrence of cluster headache in a similar sample from the general population would not be expected to be more than 1 patient, it may be safe to suggest that the specificity of the Hungarian version may be noticeably higher in representative samples.

Another issue affecting the specificity and NPV of the patients may have been a diagnostic error in chronic TTH patients. As outlined in the Results, 13 of the 19 chronic TTH patients with a positive ID-Migraine score could be considered migraineurs based on the characteristics of their headaches. As follow up data were not available for most TTH patients, this suggestion could neither be proved or disproved: however it is plausible that patients having chronic TTH and episodic migraines may not have had the latter diagnosis during their clinical visit. Our clinical experience with patients (not taking part in this study) who are followed up with a headache diary is that at least 20% of the patients who describe only migraine during the first visit would eventually be fund to have tension type headaches (TTH) as well, and a smaller percentage of patients originally diagnosed as ‘pure’ chronic TTH would also have attacks that fulfil the criteria of migraine. (This experience is not adequately reflected in the study as the number of patients included in the test-retest reliability part is still quite small.) These observations primarily affect the positive and negative predictive value and may increase the false positive test ratio, thus reducing the specificity of the questionnaire, and increasing its sensitivity and classification error.

A major limitation of our study is that our patients were recruited from patients visiting specialist headache services, so the sample is not representative of the general population. In particular, patients with episodic TTH were hugely underrepresented, and cluster headache patients were overrepresented. It is to be expected that, regardless of the diagnosis, patients with more severe head pain and accompanying symptoms would be overrepresented, and this may result in better quality indicators.

A further major limitation, that concerns the applicability of the ID-Migraine in the Hungarian population, is represented by the low specificity and low negative predictive value observed in our sample, the reasons of which are discussed above. The fact that test-retest reliability could only be tested in 10.5% of the patients represents a further limitation, although this percentage is actually slightly higher, than in the Portuguese validation study [19]. Finally, the fact that a minority of patients filled in the questionnaire after the medical visit is also a limitation, because the questions asked by the neurologist may have reinforced the patients’ memories of the characteristics of headache that are the items in the ID-Migraine questionnaire.

## Conclusion

Our validation study proved that the Hungarian version of the ID-Migraine Questionnaire is a reliable tool to screen migraine patients in Hungary, with a high sensitivity and positive predictive value. However, mainly because of the low specificity observed in the current study, using ID-Migraine as a standalone diagnostic tool in Hungarian patients is currently not feasible. Further testing of the instrument is required, preferably in a sample from the general population.

## Abbreviations

CI: Confidence interval
ICHD3-β: International Classification of Headache Disorders
MOH: Medication overuse headache
NI: No information
NPV: Negative predictive value
PPV: Positive predictive value
ROC: Receiver operating curve
SUNCT: Short-lasting unilateral neuralgiform headache with conjunctival injection and tearing
